# The HOME Study: study protocol for a randomised controlled trial comparing the addition of Proactive Psychological Medicine to usual care, with usual care alone, on the time spent in hospital by older acute hospital inpatients

**DOI:** 10.1186/s13063-019-3502-5

**Published:** 2019-08-07

**Authors:** Jane Walker, Katy Burke, Mark Toynbee, Maike van Niekerk, Chris Frost, Nicholas Magill, Simon Walker, Mark Sculpher, Ian R. White, Michael Sharpe

**Affiliations:** 1 Psychological Medicine Research, University of Oxford Department of Psychiatry, Warneford Hospital, Oxford, UK; 2 Department of Medical Statistics, London School of Hygiene and Tropical Medicine, London, UK; 3 Centre for Health Economics, University of York, Heslington, York, UK; 4 London Hub for Trials Methodology Research, MRC Clinical Trials Unit at UCL, London, UK

**Keywords:** Randomised controlled trial, protocol, psychological medicine, liaison psychiatry, multi-morbidity

## Abstract

**Background:**

Prolonged acute hospital stays are a major problem for older people and for health services. Failure to effectively manage the psychological and social aspects of illness is an important cause of prolonged hospital stays. Proactive Psychological Medicine (PPM) is a new way of providing psychiatry services to medical wards. PPM is proactive, focussed, intensive and integrated with medical care. A major aim of PPM is to reduce the time older people spend in hospital because of unmanaged psychological and social problems. The HOME Study will test the effectiveness and cost-effectiveness of PPM.

**Methods/design:**

A two-arm parallel-group randomised controlled superiority trial, with a linked health economic analysis and an embedded process evaluation, will be conducted at three sites. A total of 3588 participants will be recruited and randomised to usual care or usual care plus PPM. The primary outcome is the number of days spent as an inpatient in a general hospital in the month (30 days) post-randomisation. Secondary outcomes for each participant (measured at 1 and 3 months) include quality of life, independent functioning, symptoms of anxiety and depression, cognitive function, and their experience of the hospital stay.

**Discussion:**

The trial has been designed to produce findings that are generalisable to all older medical inpatients (including those with cognitive impairment). It will provide information on the effectiveness and cost-effectiveness of PPM, which we hope will be of value to patients, clinicians, managers and service planners.

**Trial registration:**

ISRCTN86120296. Registered on 3 January 2018.

**Electronic supplementary material:**

The online version of this article (10.1186/s13063-019-3502-5) contains supplementary material, which is available to authorized users.

## Background

Prolonged acute hospital stays are a major problem for older people and for health services. In the UK, National Health Service (NHS) acute hospitals have more than two million unplanned admissions of people aged 65 and older every year. The greater length of stay of older patients means that these admissions account for most (70%) of the available emergency bed days [1]. Excessive time in hospital is bad for patients: it leads to hospital-acquired illnesses, demoralisation and loss of independence after discharge [2]. It is also bad for the hospitals as it reduces the availability of beds for other people and increases costs. For these reasons, health services are seeking to reduce the time older people spend in hospital and to improve out of hospital care. A recent review of organisational interventions to reduce length of stay in hospital found that, whilst many of the initiatives that aimed to achieve this showed promise, none were of proven effectiveness [3].

The reasons for prolonged hospital stays include not only the complexity of older patients’ medical problems, but also inadequately managed psychological and social problems. The psychological problems include psychiatric illnesses such as delirium, dementia and depression as well as minor cognitive impairment or anxiety, all of which may slow patients’ discharge from hospital [4, 5]. The social problems include delays in organising post-discharge care arrangements, family members’ expectations or concerns about where the patient will go when they leave hospital, and miscommunications and conflicts about discharge planning within the clinical team. Failure to effectively manage these problems is well documented [6].

These psychological and social problems are usually addressed by providing a type of psychiatric care to medical wards called liaison psychiatry. Liaison psychiatry services consequently have the potential to reduce the time that older people spend in hospital. However, they currently have limited ability to do this because: (a) they operate using a referral model and therefore see only the small minority of patients identified as having obvious psychiatric problems by medical teams, (b) they do not have a consistent focus on reducing time in hospital, (c) their contributions to the care of these patients is typically limited to consultations and advice and (d) they have limited integration with the patient’s clinical team. Perhaps not surprisingly, the current evidence for the effectiveness and cost-effectiveness of such services is very limited [7].

We have developed a new service model called Proactive Psychological Medicine (PPM), which aims to be more effective in reducing time in hospital. The new model aims to address the limitations of the current approach: (a) it is proactive in seeing all admitted patients (building on the experience of a proactive psychiatric consultation service initiated in Yale Newhaven Hospital in the USA [8, 9]), (b) it takes a broad biopsychosocial approach focussing on facilitating prompt discharge, (c) it provides an intensive contribution to care with comprehensive consultant assessment and daily follow-up and (d) it is integrated, with PPM clinicians working as members of the patient’s extended medical team. We have piloted this new PPM service model and found it to be both feasible and acceptable in an NHS general hospital setting.

The HOME Study aims to determine whether adding PPM to usual care reduces the time spent by older patients in acute hospital wards in the month (30 days) after randomisation (primary outcome), when compared with usual care alone. A number of secondary outcomes, including patients’ views of their length of time in hospital and their quality of life will also be evaluated. We will also determine the cost-effectiveness of adding PPM to usual care.

## Methods/design

### Design

**Fig. 1:**
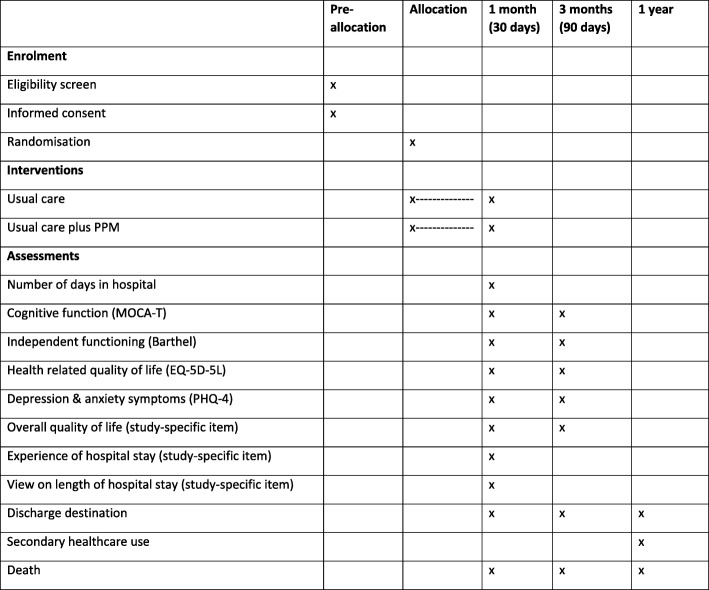
The HOME Study: schedule of enrolment, interventions and assessments. MOCA-T Montreal Cognitive Assessment, telephone version, PHQ-4 four-item Patient Health Questionnaire, PPM Proactive Psychological Medicine

This is a pragmatic multicentre two-arm parallel-group randomised controlled superiority trial with a linked health economic analysis and an embedded process evaluation. Figure 1 lists the schedule of enrolment, interventions and assessments.

### Patients

**Fig. 2:**
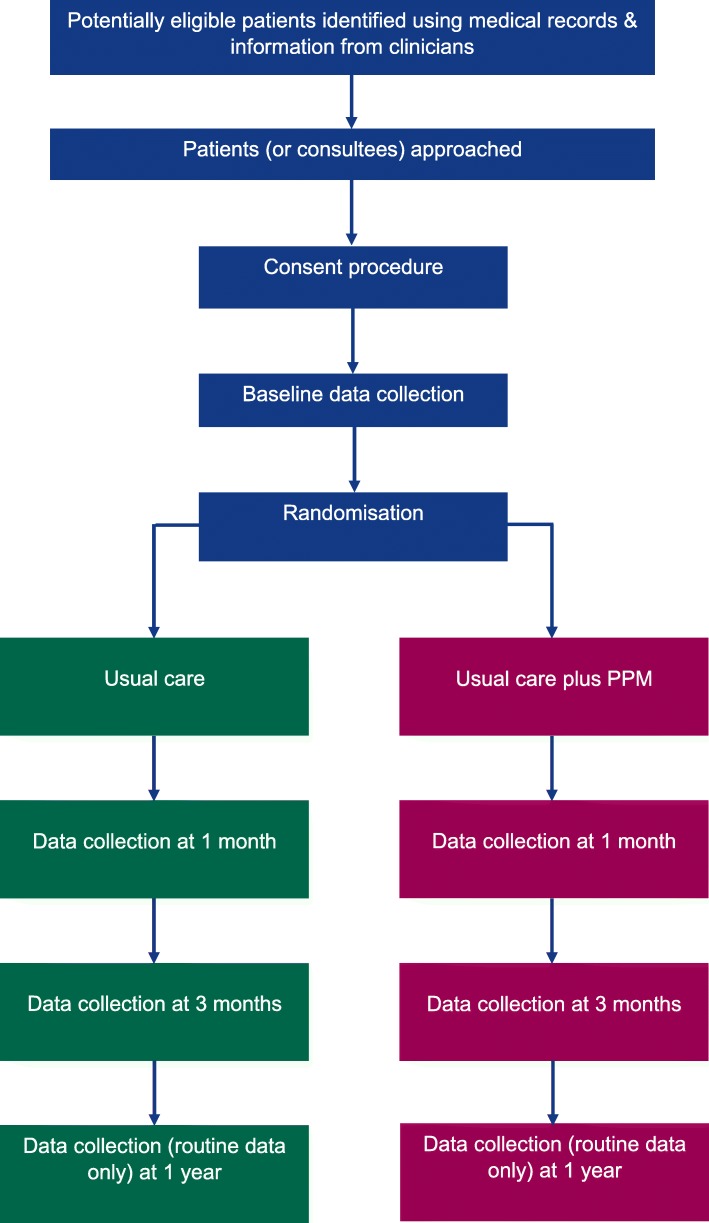
Flow of patients through the study

Three thousand five hundred eighty-eight patients will be recruited from the acute wards (not emergency departments) of Oxford University Hospitals NHS Foundation Trust, Royal Devon and Exeter NHS Foundation Trust and Cambridge University Hospitals NHS Foundation Trust (see Figure 2). We aim to recruit from at least four wards per hospital over at least 18 months.

To be included in the trial, patients must:

- be aged 65 or older.
- be an inpatient in an acute ward where trial recruitment is taking place.
- have been admitted non-electively (i.e. their hospital admission was unplanned).
- be expected by their clinical team to remain an inpatient for at least 2 days from the time of trial enrolment.
- be able to give informed consent or if unable to give consent, a consultee advises that trial participation is appropriate.

Patients will be excluded if at the time of enrolment:

- they are moribund, which is defined for this trial as when the clinicians caring for a patient estimate that they are likely to die before discharge from hospital.
- their participation in the trial is judged to be clinically or practically inappropriate (e.g. the patient is not from the local area served by the hospital).
- they have already been enrolled in the trial.
- they have already been referred to the usual care liaison psychiatry team.
- they have already been a general hospital inpatient continuously for 1 week.
- they do not read or speak English.

### Patient identification and enrolment

Screening will be used to identify potential participants, in order to obtain a representative sample of the relevant population and to give all potentially eligible patients the opportunity to participate. Researchers will screen all patients admitted to the participating wards during the trial period for eligibility. This will be done by accessing their medical records and also obtaining relevant information from clinicians. Patients identified as eligible by this process will be offered both verbal and written information about the trial. They will be given a full explanation of both treatment allocations, and the procedures for randomisation and outcome data collection. Written informed consent will then be obtained for trial participation (procedures for patients who lack capacity are described below). At all stages, the research team will endeavour to record reasons for non-participation.

### Recruitment of patients who lack capacity

"Capacity" refers to a patient’s ability to make the decision whether to participate in the HOME Study. Recruitment of patients who lack capacity will be in accordance with the Mental Capacity Act 2005 with specific reference to sections 30 to 34. A personal consultee (a family member, a carer, a friend, an attorney under a Lasting Power of Attorney or a court-appointed deputy provided that they had a relationship with, or personal knowledge of, the person lacking capacity before their appointment as deputy) will be identified for the patient where possible. The personal consultee will be asked to advise on the patient’s likely thoughts and feelings about the research and whether they should be enrolled in the trial. If a personal consultee cannot be identified or cannot be contacted within 24 h, a nominated consultee will be approached for advice regarding the patient’s participation in the trial.

### Baseline data

The following baseline data will be collected:

- name of hospital and ward at the time of recruitment.
- NHS and hospital numbers (to allow matching with routine data).
- date of birth.
- sex.
- ethnicity.
- relationship status (whether the patient has a partner or spouse).
- usual place of residence (private home, care home etc.).
- postcode (to calculate the deprivation index and to determine whether they live in an urban or rural setting).
- whether the participant lives alone.
- employment status.
- reason for hospital admission (presenting complaint or working diagnosis).
- diagnoses (medical and psychiatric) recorded on admission.
- medication prescribed.
- date of hospital admission.
- date of admission to specified acute ward.
- days in hospital prior to enrolment.
- cognitive function, measured by the Montreal Cognitive Assessment, telephone version [10].
- independent functioning, measured by the Barthel Index of Activities of Daily Living [11].
- health-related quality of life, measured by the EQ-5D-5L [12].
- symptoms of anxiety and depression, measured by the Patient Health Questionnaire-4 [13].
- overall quality of life, measured by a trial-specific item.
- secondary healthcare use (including number of admissions to hospital) in the year prior to randomisation.

Questionnaire data will be collected from the participant using a brief face-to-face interview as soon as possible prior to randomisation. Some participants will be unable to give reliable data, even with help. In this instance, data will be collected from proxies wherever possible.

### Randomisation

A database software algorithm, designed by the trial statistician, will allocate participants to usual care plus PPM or usual care alone in a 1:1 ratio with stratification by putative prognostic variables: hospital, sex and age (65–74, 75–84, and ≥85). The algorithm is based on Stata’s “ralloc” command and utilises random permuted blocks of variable size. The required random seed was selected by the Oxford Clinical Trials Research Unit, which will implement the randomisation system. The participant’s details will be entered into the database via a secure website.

### Blinding

Trial statisticians and research staff who collect outcome data will be blinded to participants’ allocated interventions. HOME Study researchers who recruit participants will carry out the randomisation procedure described above. They will inform participants of their treatment allocation and will inform the PPM teams about participants who have been allocated to usual care plus PPM. Recruiting researchers, participants and clinicians will not be blinded to treatment allocation.

### Trial treatment: intervention

The intervention is usual care supplemented with PPM, which has four main components:

1. Early proactive biopsychosocial assessment of newly admitted patients using a biopsychosocial approach to identify all problems, including psychiatric illness.
2. The creation of a systematic management plan to address those problems that pose potential barriers to prompt discharge.
3. Implementation of the management plan with daily progress reviews.
4. Integrated working with ward teams (doctors, nurses, allied health professionals and social care professionals) and out of hospital services to ensure that the management plan is implemented.

PPM will be delivered at each trial site by a specially trained consultant in psychological medicine/liaison psychiatry and an assisting clinician, who will work as additional members of the patient’s medical team (the assisting clinician may be a junior doctor, a nurse or an allied health professional with experience of working in psychological medicine/liaison psychiatry). Each of these clinicians will have a backup to cover leave. To ensure fidelity to the service model, the PPM clinicians will: (a) deliver PPM according to a service manual, (b) use a PPM checklist for each patient, (c) be required to pass quality assessments prior to treating trial participants, (d) participate in weekly joint supervision by video-conference and (e) undergo regular quality assurance checks throughout the trial.

### Trial treatment: usual care

This is a pragmatic trial and the comparator arm is usual care. Participants allocated to this arm will receive usual medical care, including the option for the patient’s medical team to request a consultation from the hospital’s usual liaison psychiatry team. Referrals to usual care liaison psychiatry will be recorded (see Process evaluation below).

### Primary outcome

The primary outcome is the number of days spent as an inpatient in a general hospital in the month (30 days) post-randomisation.

### Secondary outcomes

The following secondary outcomes will be assessed:

- cognitive function, measured by the Montreal Cognitive Assessment, telephone version, at 1 and 3 months post-randomisation [10].
- independent functioning, measured by the Barthel Index of Activities of Daily Living at 1 and 3 months post-randomisation [11].
- health-related quality of life, measured by the EQ-5D-5L at 1 and 3 months post-randomisation [12].
- symptoms of anxiety and depression, each measured by the relevant two items of the Patient Health Questionnaire-4 at 1 and 3 months post-randomisation [13].
- overall quality of life, measured by a trial-specific item (0 to 10 scale) at 1 and 3 months post-randomisation.
- patient’s experience of hospital stay, measured by a trial-specific item (0 to 10 scale) at 1 month post-randomisation.
- patient’s view on the length of their hospital stay, measured by a trial-specific item at 1 month post-randomisation.
- discharge destination.
- secondary healthcare use in the year post-randomisation (including total length of index admission, number of readmissions and number of days in hospital).
- death in the year post-randomisation.

### Measures of cost and health-related quality of life

The following economic outcome measures will be assessed:

- quality adjusted life years (QALYs), estimated using the EQ-5D-5L measure.
- cost of secondary healthcare use.
- cost of PPM.

### Outcome data collection

Data describing the participant’s hospital stay, their discharge destination, subsequent hospital admissions, secondary healthcare use and mortality data will be obtained from national datasets of routinely collected clinical data and from local hospital records and datasets. At 1 month (30 days) and 3 months (90 days) post-randomisation, a member of the research team will contact the participant (or an appropriate proxy) to administer the questionnaires by telephone or face to face. The time windows for data collection are as follows: 1 month data will be collected between day 30 and day 75 post-randomisation (inclusive of these dates) and 3 month data will be collected between day 90 and day 135 post-randomisation (inclusive of these dates).

Active measures will be taken to minimise missing data. These will include:

- using routinely collected clinical data to provide the primary outcome.
- obtaining full contact details from participants.
- obtaining a back-up best contact address (i.e. contact details of a friend or relative nominated by the participant).
- recording participants’ discharge destination from hospital.
- collecting data from proxies where participants are unable to give reliable data.
- reminder telephone calls and letters.
- checks with the patient’s general practice to determine if they are alive and/or have moved address.

### Data management

To ensure that all data are reliable and have been processed correctly, standard operating procedures will be implemented at each stage of the data handling process and all electronic data will be checked for accuracy as follows: 100% check for the primary outcome measure and a random minimum 10% sample check for all other outcome measures.

Personal data will be stored separately from research data, once transferred to the main trial office. All documents will be stored securely and only accessible by trial staff and authorised personnel. Data will be anonymised as soon as it is practical to do so.

### Safety

The Serious Adverse Events (SAEs) which will be recorded and reported in this trial are deaths by any cause in the 30 days post-randomisation. Re-hospitalisations, life-threatening illness and significant disability are to be expected in this group of patients and will not, therefore, be recorded as SAEs.

### Sample size

A total of 3588 participants is required to detect a reduction of 1 day (from 9 to 8 days, standard deviation 9 days) in mean number of days in hospital with 90% power at the 5% significance level using a two-tailed test and allowing for 5% loss to follow-up.

### Statistical analyses

A single main analysis will be performed at the end of the trial when all outcome data have been collected. A detailed statistical analysis plan will be developed prior to closure of the trial database and prior to the unblinding of the treatment allocations. The main analysis of the primary and secondary outcomes will follow the intention-to-treat principle (i.e. the participants will remain in the group they were randomised to and not analysed according to the interventions actually received). For the primary outcome (number of days spent in hospital in the 30 days post-randomisation), the difference between the means with a 95% confidence interval will be reported. This will be obtained from a linear regression model. This model will include: (a) centre (Cambridge, Exeter or Oxford) by treatment interaction terms, (b) stratification factors (hospital, sex and age; which will be treated as continuous in the analysis model, but in three categories for stratification) as fixed effects and (c) wards as either fixed or random effects (the final choice being dependent on the number of wards included). The primary outcome will be a weighted mean of the three centre-specific treatment effects, with weights proportional to the number of people randomised at each centre. As a check on the robustness of results to normality assumptions, non-parametric bootstrap (bias corrected and accelerated, 2000 replications, with allowance for stratification) methodology will be used to construct the confidence interval. Secondary continuous outcomes will be analysed in an analogous fashion to the primary outcome. For binary outcomes, odds ratios will be estimated. These will be obtained from logistic regression models (with adjustment for stratification factors). The effect measure will be the exponent of the weighted means of the three centre-specific log odds ratios, reported as an adjusted odds ratio. Further secondary analysis will consider time until leaving hospital as a survival time, with adjusted Cox models used to estimate hazard ratios. The effect measure will be the exponent of the weighted means of the three centre-specific log hazard ratios, reported as an adjusted hazard ratio. 

### Economic evaluation

Cost-effectiveness will be assessed from the perspective of the NHS with outcomes expressed in terms of QALYs, in line with current UK guidance for economic evaluations [14]. If one form of management is more costly and more effective, incremental cost-effectiveness ratios will be presented for the alternative options and compared with appropriate cost-effectiveness thresholds for the NHS. These will also be presented as net health effects with thresholds representing the forgone opportunities to improve other patients’ health (opportunity costs) [15]. For the base case, cost-effectiveness will be assessed over the 1-year trial period. The within-trial analyses will be conducted using appropriate statistical techniques to control for any baseline differences in covariates between patient groups and for issues with non-normality of cost and outcome data [16]. Missing data will be handled using imputation with chained equations [17]. Decision uncertainty resulting from estimating the within-trial analysis cost-effectiveness will be presented using cost-effectiveness acceptability curves [18]. The consequences of decision uncertainty and the potential value of additional research will be assessed using value of information analysis [19]. Scenario and sensitivity analyses will also be undertaken to examine the impact of key assumptions and uncertainties. If important differences in costs and/or outcomes between the management strategies are found over the trial period and would be expected to persist over the longer term, extrapolation of the trial results will be conducted. This will involve the development of a decision analytic model, which will synthesise evidence from the trial with other external sources to estimate the costs and QALYs over each patient’s lifetime [19, 20].

### Process evaluation

An embedded process evaluation will be used to describe: (a) the relevant care received by participants during their hospital stay, (b) patients’, carers’ and healthcare professionals’ experience of PPM and (c) the context in which PPM is delivered during the trial. Data will be collected from participants’ medical records and through qualitative interviews with participants (a subgroup of the total sample), carers and healthcare professionals who deliver PPM or work on the relevant hospital wards.

### Trial management and monitoring

The Trial Management Group (TMG) will be responsible for the day-to-day running of the trial, including monitoring recruitment and outcome data collection, and communicating protocol changes to the relevant parties. The trial will be overseen by an independent Trial Steering Committee (TSC), which will meet at least annually to consider and address strategic issues. A Data Monitoring Committee (DMC), members of which will act independently of the TSC , TMG and funder, will monitor data and make recommendations to the TSC on whether there are any ethical or safety reasons why the trial should not continue. The DMC will monitor the occurrence of SAEs and, if unblinded, suspected unexpected serious adverse reactions, i.e. serious adverse events that are likely to be due to the implementation of PPM. The DMC will focus on the number of participant deaths that occur within 30 days of trial enrolment. Interim analyses of the primary outcome data will not be undertaken because these require data that will not be available during the relatively short recruitment period. There are, therefore, no statistical stopping rules for this trial related to the primary outcome and the DMC will recommend stopping only on safety grounds. Audits appropriate to the trial will be planned and conducted by the Oxford Clinical Trials Research Unit.

### Dissemination

The results of the trial will be analysed and published as soon as possible. The results will be reported in the first instance to the funding body and study collaborators. A writing committee, chaired by the chief investigator, will be constituted with the aim of prompt publication of trial reports in high-impact journals. A lay summary of the trial findings will be made available on the trial website.

## Discussion

This trial addresses an important and topical question: does addressing older medical patients’ psychological and social problems with a new psychiatry service model reduce the time they spend in acute hospitals and does it produce better patient outcomes?

The trial has been designed with the aim of providing a clear answer to this question. To ensure that the findings are robust we will: (a) recruit a large enough sample to detect a clinically meaningful effect if one exists, (b) recruit a representative sample by using screening to identify potential participants, including patients with cognitive impairment, and by recruiting in hospitals that serve urban and rural populations of varying socioeconomic status, (c) deliver the experimental intervention with adequate quality assurance whilst taking steps to minimise contamination of usual care, (d) evaluate effectiveness using a primary outcome that is not susceptible to reporting bias or missing data, supplemented with patient-reported secondary outcomes, (e) conduct an embedded process evaluation so that if PPM is found to be effective we have information on how best to implement it and if it is found to be ineffective we have information on the possible reasons for this finding  and (f) undertake a cost-effectiveness analysis to establish whether PPM can be considered a good use of resources compared to other NHS activities.

A major consideration in the design of this trial was whether to use cluster or individual randomisation. Cluster randomisation was considered on the basis that PPM teams work in an integrated way with patients' other hospital clinicians and there is a potential for usual care to be contaminated by elements of PPM. However, we concluded that individual randomisation was most suitable because:

- PPM is designed to affect patient care at the individual level and delivered with this in mind (e.g. PPM teams work in collaboration with other clinical staff but do not provide formal education or seek to change the way a ward operates).
- Contamination is likely to be minimal because PPM is so dissimilar to traditional liaison psychiatry consultations that participants allocated to usual care would only receive it if a major change were to occur in the configuration of existing services.
- There is no clearly appropriate natural cluster (e.g. a ward) because hospitals are organised differently. Some have ward-based medical teams, whereas others have teams that are responsible for patients admitted during the course of a given period (on-take teams).
- If we randomised wards, these would be open clusters. The ward’s allocation to PPM or usual care would be known to clinical staff and might influence which patients they admitted to each ward.

We have, therefore, elected to use individual randomisation and to take precautions to limit contamination, including ensuring that ward teams understand the need to adhere to randomised patient allocation and the separation of PPM teams from those delivering liaison psychiatry services as part of usual care.

We also carefully considered which measures should be used. The primary outcome uses routinely collected data and therefore neither places a burden on participants nor depends on their ability to respond to questions from a researcher. We conducted pilot work to ensure the secondary outcome measures would be suitable for unwell older people who may have cognitive impairment. They were chosen for their suitability to be delivered by telephone or face to face, and to proxies when participants are unable to provide data.

The trial aims to provide robust information on the role of psychiatry in the care of older medical inpatients, which we hope will be of value to patients, clinicians, managers and service planners.

### Trial status

Recruitment commenced on 2 May 2018. Recruitment is expected to be completed by 31 March 2020. The current protocol version is 6.0, dated 9 November 2018.

## Additional file

Additional file 1: SPIRIT 2013 Checklist: Recommended items to address in a clinical trial protocol and related documents. (DOCX 51 kb)

## Data Availability

Not applicable.
